# The extensive transgenerational transcriptomic effects of ocean acidification on the olfactory epithelium of a marine fish are associated with a better viral resistance

**DOI:** 10.1186/s12864-022-08647-w

**Published:** 2022-06-17

**Authors:** Mishal Cohen-Rengifo, Morgane Danion, Anne-Alicia Gonzalez, Marie-Laure Bégout, Alexandre Cormier, Cyril Noël, Joëlle Cabon, Thomas Vitré, Felix C. Mark, David Mazurais

**Affiliations:** 1 IFREMER, PFOM-ARN, 29280 Plouzané, France; 2 Ploufragan-Plouzané Laboratory, Fish Viral Pathology Unit, French Agency for Food, Environmental and Occupational Health and Safety (ANSES), Technopôle Brest-Iroise, 29280 Plouzané, France; 3 MGX, CNRS, INSERM, University of Montpellier, Biocampus Montpellier, Montpellier, France; 4 MARBEC, University of Montpellier, CNRS, IFREMER, 34250 Palavas-les-Flots, IRD France; 5 IFREMER, SEBIMER, 29280 Plouzané, France; 6 Alfred Wegener Institute Helmholtz Centre for Polar and Marine Research (AWI), Department of Integrative Ecophysiology, 27570 Bremerhaven, Germany

**Keywords:** Anti-viral immunity, Betanodavirus, Climate change, European sea bass, Long-term transgenerational ocean acidification, Metabolism, Neuro-sensory system, Olfactory epithelium, Transcriptomics

## Abstract

**Background:**

Progressive CO_2_-induced ocean acidification (OA) impacts marine life in ways that are difficult to predict but are likely to become exacerbated over generations. Although marine fishes can balance acid–base homeostasis efficiently, indirect ionic regulation that alter neurosensory systems can result in behavioural abnormalities. In marine invertebrates, OA can also affect immune system function, but whether this is the case in marine fishes is not fully understood. Farmed fish are highly susceptible to disease outbreak, yet strategies for overcoming such threats in the wake of OA are wanting. Here, we exposed two generations of the European sea bass (*Dicentrarchus labrax*) to end-of-century predicted pH levels (IPCC RCP8.5), with parents (F1) being exposed for four years and their offspring (F2) for 18 months. Our design included a transcriptomic analysis of the olfactory rosette (collected from the F2) and a viral challenge (exposing F2 to betanodavirus) where we assessed survival rates.

**Results:**

We discovered transcriptomic trade-offs in both sensory and immune systems after long-term transgenerational exposure to OA. Specifically, RNA-Seq analysis of the olfactory rosette, the peripheral olfactory organ, from 18-months-old F2 revealed extensive regulation in genes involved in ion transport and neuronal signalling, including GABAergic signalling. We also detected OA-induced up-regulation of genes associated with odour transduction, synaptic plasticity, neuron excitability and wiring and down-regulation of genes involved in energy metabolism. Furthermore, OA-exposure induced up-regulation of genes involved in innate antiviral immunity (pathogen recognition receptors and interferon-stimulated genes) in combination with down-regulation of the protein biosynthetic machinery. Consistently, OA-exposed F2 challenged with betanodavirus, which causes damage to the nervous system of marine fish, had acquired improved resistance.

**Conclusion:**

F2 exposed to long-term transgenerational OA acclimation showed superior viral resistance, though as their metabolic and odour transduction programs were altered, odour-mediated behaviours might be consequently impacted. Although it is difficult to unveil how long-term OA impacts propagated between generations, our results reveal that, across generations, trade-offs in plastic responses is a core feature of the olfactory epithelium transcriptome in OA-exposed F2 offspring*,* and will have important consequences for how cultured and wild fish interacts with its environment.

**Supplementary Information:**

The online version contains supplementary material available at 10.1186/s12864-022-08647-w.

## Background

Atmospheric CO_2_ concentration has increased from preindustrial levels of 280 ppm to the current value of 414 ppm [1], and is expected to reach ~ 1 000 ppm by the end of this century [2–4]. These changes lead to ocean acidification (OA) adn, include changes in the carbonate system equilibrium such as reduced carbonate ion concentrations and pH. According to the future warming scenario RCP 8.5, seawater pH will decrease by 0.3–0.4 units by 2100 in the North Atlantic Ocean [2, 3, 5].

OA alone or coupled with ocean warming poses global-scale threats to marine life ranging from the invisible scale of microbes to ecosystem levels [6–9]. Given their efficient capacity for maintaining acid–base homeostasis, fishes have long been considered to be robust organisms capable of tolerating OA [10–13]. However, reported indirect impacts of OA include both inter- and intra-specific changes in sensory behaviours [14], which result from directly altered neurosensory systems [15–23] that ultimately affect reproduction, fitness and mortality [24–27]. Although such effects are complex and vary with both biological traits and methods employed [28], sensory behavioural alterations induced by acid–base regulation are generally explained as a GABAergic system dysfunction due to an inversion of the Cl^−^/HCO_3_^−^ channels across neuronal membranes [24, 29]. However, molecular and electrophysiological approaches also suggest that changes in calcium homeostasis and decrease in synaptic plasticity underlie OA impacts on synaptic activity in the olfactory bulb [30]. Likewise, a possible additional mechanism by which OA-induced protonation lead to changes in the charge distribution of odorants and their receptors in the olfactory epithelium suggests that OA might directly alter olfaction in marine fish [31, 32].

The capacity of fish to maintain the functioning of their neurosensory system in an acidic environment is mediated by both the parental environment [33] and the offspring potential to acclimate ﻿through plastic responses that are susceptible to vary across generations [34–39]. ﻿Transgenerational epigenetic inheritance is therefore the mechanism by which a change in the parental environment shapes the plastic phenotypical responses of their offspring [34, 36, 38]. For instance, parental acclimation to OA in anemonefish and ﻿three-spined sticklebacks increased growth and survival of offspring under elevated CO_2_ [35, 37]. ﻿Transgenerational exposure to OA is therefore crucial to understand how species would cope near-future OA across multiple generations.

Relative to the well-recognized influence of ocean warming on the emergence of pathogenic species and spread of viral diseases [9, 40], the influence of OA has received less attention, although it is known that virus abundance and virus-host interactions are altered by OA [41–43]. Even though the environment influences disease spread, it can also modulate the host immune responses to mitigate infection. Understanding how the environment, namely OA, influences the host immune system, and more specifically the sensitivity to pathogens, will become critical for predicting future changes in both population dynamics and aquaculture.

Earlier studies reported that OA can alter immune system parameters in marine invertebrates [44–51]. Much less is known in marine fishes [52, 53], and especially species of ecological and commercial importance, such as the European sea bass (*Dicentrarchus labrax*). Recently, the *cbln11* gene, which is involved in the pathogen defence, was reported to be up-regulated in the olfactory rosette of two successive generations of *D. labrax* exposed to OA, suggesting that OA can induce immune system changes [54]. As the olfactory epithelium is in constant contact with seawater, it functions as a gateway for invading pathogens [55] and, as an arm of the mucosal immune system, capable of eliciting a strong and rapid antiviral response [56, 57]. However, although a transcriptomic analysis of the olfactory bulb of *D. labrax* after seven days of exposure to OA within a generation detected significant expressions changes in genes involved in synaptic development, growth and ion transport, genes involved in the immune system were not significantly altered [30].

Here, we reasoned that longer-term exposure to OA would serve as a proxy for *D. labrax* acclimation to OA, and that a transcriptome analysis of the olfactory epithelium from F2 offspring would help us understand, at a molecular level, how fish acclimate over several years and across two successive generations. We also asked how OA-induced plastic responses impacts pathogen susceptibility by conducting an experimental betanodavirus challenge. Betanodavirus infections in *D. labrax* occur with high frequency at every life stage in aquaculture facilities [58–60] and causes viral nervous necrosis (VNN) by infecting the brain and retina, resulting in mass mortality and a range of neurological and behavioural abnormalities [58, 61]. As betanodavirus can infect more than 120 species of marine and freshwater fishes as well as invertebrates [62], an outbreak represents a significant risk for breeding, rearing, and harvesting of fish in all types of water environments.

## Results

**Fig. 1:**
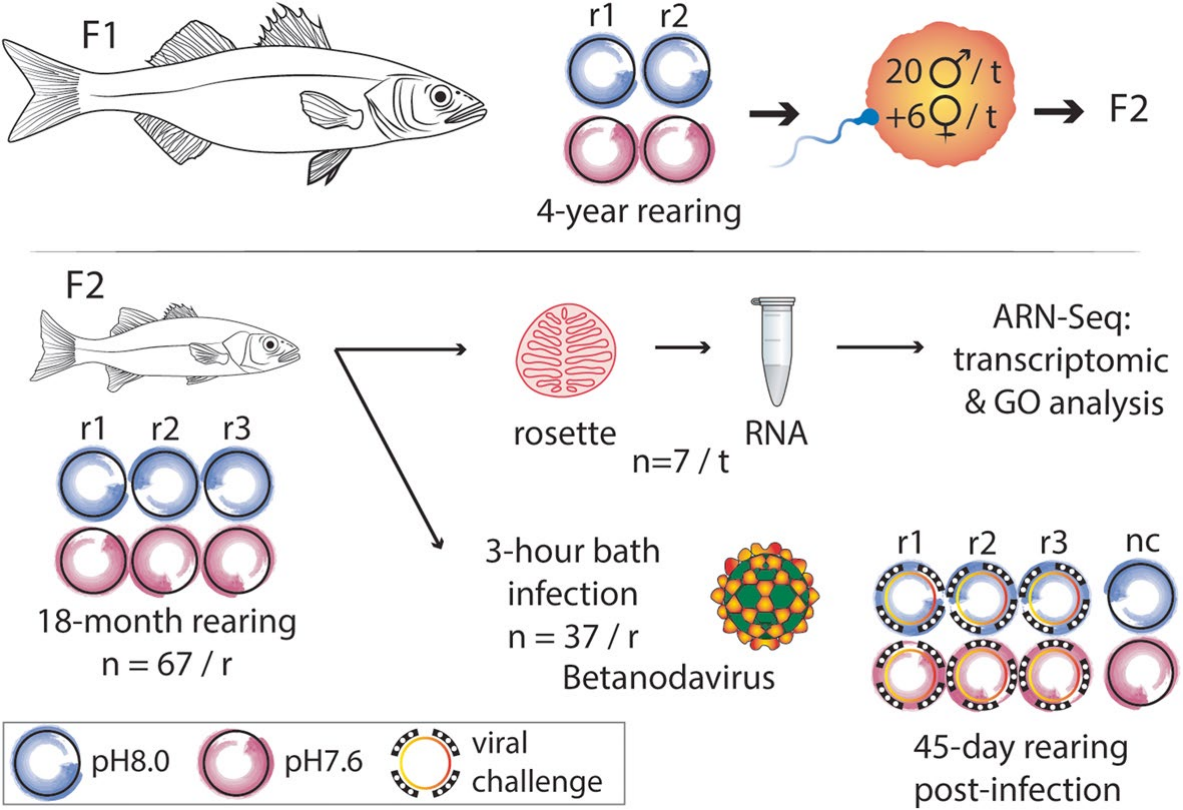
Methodological summary. Rearing times and procedures applied on *Dicentrarchus labrax* parental linage (F1) and their offspring (F2) exposed to either the control (pH8.0, blue) or the acidified (pH7.6, magenta) treatment. *RNA* ribonucleic acid, *r* replica

Here, we designed a long-term experimental paradigm in which parental *Dicentrarchus labrax* (the F1 generation), were reared in the same pH conditions as their offspring (the F2 generation) (control treatment pH8.0 or OA treatment pH7.6, as predicted by 2100 in the IPCC RCP8.5 scenario). To generate the F2 generation, we collected gametes from 4-year-old F1 fish that had been reared from larval stage to adulthood in control or OA conditions [54, 63–66]. For each pH treatment, we collected and pooled sperm and eggs from 20 males and 6 females (see methodological summary in Fig. 1). We then performed in vitro fertilization, after which the resulting F2 generation was returned to OA or control conditions [54, 63–66] (Fig. 1). After 18 months, we split our experimental F2 cohorts into two experimental arms; one in which we performed gene expression profiling of olfactory rosette tissue (7 each from control and OA rearing conditions, Fig. 1), and a second arm in which we conducted a betanodavirus challenge to investigate how long-term OA conditions impact on viral infection parameters in *D. labrax* (Fig. 1).

### Viral challenge

**Fig. 2:**
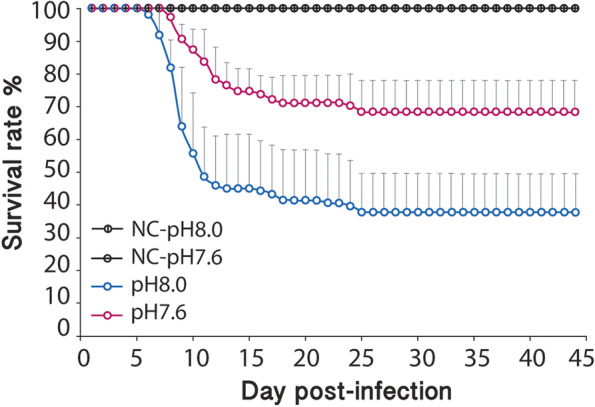
Survival rate (% + sd) of F2 *Dicentrarchus labrax* after viral challenge. Curves indicate the survival rate of non-infected juveniles (black) and infected juveniles with W80 strain either from the control treatment (pH8.0, blue) or the acidified (pH7.6, magenta) treatment. Each challenge was performed in triplicate with 37 fish per replica tank. Survival was monitored during 45 days post-infection. *NC,* negative control

In non-infected fish, and as expected, we did not detect any mortality, clinical symptoms or lesions. In infected fish, symptoms of VNN disease i.e. darkening of the body, whirling swimming and hyperactivity, were evident after five days in the control treatment (dpi 5, pH8.0) but appeared three days later in fish exposed to the OA treatment (dpi 8, pH7.6). Similarly, mortality onset was staggered such that fish started dying at 7 dpi in the control treatment and at 9 dpi in the OA treatment, but after 25 days, no additional mortality was observed (Fig. 2). Survival curves were influenced by a significant tank effect in the control, but not in the OA treatment. Therefore, survival rate in pH7.6 was pooled and compared to each pH8.0 replica, and revealed to be significantly different for 2 replica tanks (*p*-values_Log rank_ = 0.0001; 0.03; 0.7). Furthermore, at the end of the challenge, more fish survived infection in OA relative to control conditions, as reflected by a final higher survival rate in OA treatment (68%) than in the control (38%) (Fig. 2). At the peak of mortality, the presence of the virus in the pool of organs (brain and ayes) was indistinguishable between treatments. At 45 dpi, no virus was detected in survivor fish, though 91.3% of them were seropositive in the control whereas only 53.8% in the OA treatment.

### Global gene expression profiling

After a 24 h fasting period, we dissected out the olfactory rosette from F2 juveniles (7 each, reared for 18 months in either OA or control conditions), extracted RNA and verified RNA integrity as described in Materials and Methods. cDNA library construction for the 14 samples and RNA-sequencing was performed by the GenomiX sequencing platform (MGX, Montpellier, France). Across the 14 libraries, Illumina sequencing generated a total of 772 449 468 reads with an average of 55 450 000 reads per library (Additional file 1). More than 90% of the sequencing reads for each library (except for one sample that was 72%) could be mapped to *D. labrax* reference genome. Of the total 26 721 transcripts, 9 112 were differentially expressed (with an adjusted *p*-value ≤ 0.01), with 4 515 (49.6%) up-regulated and 4 598 (50.4%) down-regulated in the OA-exposed cohort (listed in Additional file 2).

### Regulation of pH, bicarbonate transport and chloride homeostasis

Overall, we found that genes involved in regulation of pH, bicarbonate transport and chloride homeostasis were significantly differentially expressed (Table 1, Additional file 3). Among the genes involved in pH regulation, members of the slc9a family genes were either up-regulated (*slc9a1, slc9a7*) or down-regulated (*slc9a2, slc9a9*) in OA-reared F2 juveniles whereas genes involved in bicarbonate transport, all slc4a family members, were up-regulated. Twenty-seven chloride transport-associated gene transcripts were up-regulated by OA exposure, of which the majority (22 transcripts) included Slc12a family genes (K-Cl cotransporters) and GABA receptors.

**Table 1:** Genes involved in the regulation of pH, bicarbonate transport and chloride transport regulated (*p*-value ≤ 0.01) by ocean acidification in the olfactory rosette of F2 European Sea Bass (*Dicentrarchus labrax*) juveniles

GO term	Regulation by OA	Number of transcripts	Regulated genes
**Regulation of pH**	Up	3	*slc4a10, slc9a1, slc9a7*
	Down	4	*cahz, edn1, slc9a2, slc9a9*
**Bicarbonate transport**	Up	3	*slc4a7, slc4a8, slc4a10*
**Chloride homeostasis**	Up	22	*ano1, ano3, ano5, ano8, cftr, clcn2, clcn6, clcn7, clic4, clic5, gabra2, gabra4, gabrg2, gabrr2, glrb, slc4a10, slc12a2, slc12a4, slc12a5, slc12a7, slc12a10*
	Down	5	*abcc4, clcn5, fam131a, icln*

Several transcripts may originate from the same gene. *GO* Gene Ontology

### Neuronal plasticity and activity

**Fig. 3:**
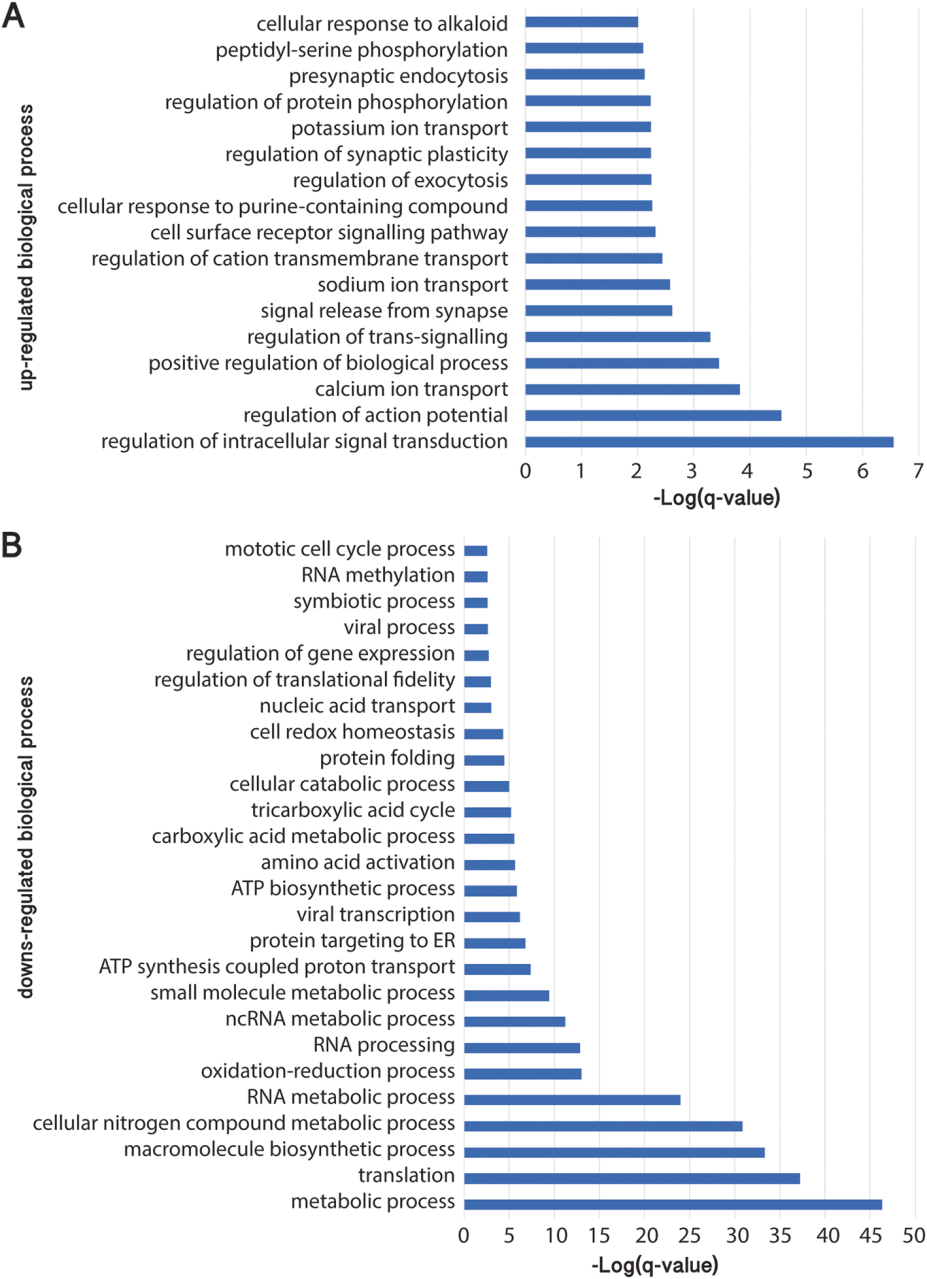
Gene ontology enrichments for biological processes. Enriched (q-value ≤ 0.01) biological processes related to the **A** up-regulated and **B** down-regulated genes in the olfactory epithelium from F2 *Dicentrarchus labrax* exposed to ocean acidification relative to controls

As predicted from an expression analysis of olfactory epithelium, GO analysis identified enrichment of up-regulated genes related to neuronal cell structures in OA-treated tissue (e.g. “neuron projection”, “synapse”, “postsynaptic density”, “voltage-gated sodium channel complex”, “neuronal cell body”) (Additional file 4). For instance, within the 90 genes related to “neuron projection”, we found axonal function associated genes such as kinesin heavy chain isoform 5c-like (*kif5c*), semaphorins, ephrin type-b receptor 2 (*ephb2*), cyclin-dependent kinase 5 (*cdk5*), amyloid beta a4 precursor protein-binding family b member 1-like (*apbb1*) and growth associated protein 43 (*gap43*) (Table 2, Additional file 5). Based on biological process GO enrichment, we found that genes associated with “signal transduction” (516 transcripts), including “regulation of small GTPase mediated signal transduction” (95 transcripts) or “cell surface receptor signalling pathway” (160 transcripts), were over-represented with OA-induced up-regulated genes (Fig. 3A, Additional file 6). Among the 187 genes involved in “intracellular signal transduction”, we found known neuronal plasticity pathways such as the Neurotrophin TRK receptor signalling pathway (19 transcripts), the Glutamate signalling pathway (13 transcripts) and the Ephrin signalling pathway (9 transcripts) (Table 2, Additional file 7). Genes related to “regulation of synaptic plasticity” (22 transcripts) were also enriched within OA-induced up-regulated genes (Fig. 3A, Additional file 6), and genes coding for calcium calmodulin-dependent protein kinase type ii subunit beta-like (*camk2b*), synaptogyrin-1 (*syngr1*) and neuroplastin-like (*nptn*) proteins exhibited significantly elevated gene expression levels (Additional file 8). In addition, biological processes related to “regulation of action potential”, were also over-represented in OA-treated tissue (Fig. 3A, Additional file 6) included genes involved in potassium, sodium and calcium ion transport (e.g. *scn2b, scn1a, cacna1d, kcn2, kcna5*) (Table 2, Additional file 9). Consistently, “regulation of neurotransmitter levels” genes were enriched within genes up-regulated by OA, including genes involved in the GABAergic signalling pathway (up-regulated: *cacna1a*, *gabra2*, *nf1, slc6a13*) (Table 2, Additional file 10).

**Table 2:** Examples of enriched gene ontologies and associated genes involved in neural plasticity and activity that were up-regulated (*p* ≤ 0.01) by ocean acidification in the olfactory rosette of F2 European Sea Bass (*Dicentrarchus labrax*) juveniles

GO term	Subcategory	Number of transcripts	Examples of regulated genes
**Neuron projection**	Axon guidance	15	*apbb1, cdk5, epha5, epha8, ephb2, kif5c, gap43, sptbn4, tgfb2*
	Axon extension	3	*cdk5, map1b, sema3a*
**Intracellular signalling transduction neuroplasticity**	Neurotrophin TRK receptor sig. pathway	19	*adcy6, arhgef7, calm1, erbb4, gab1, kras, ngef, shc1, vav2*
	Glutamate signalling pathway	13	*gnaq, glur2b, gria1a, gria3b, gria4k, grik2, grik5, grin3a, grind2d*
	Ephrin signalling pathway	9	*chn1, efna5, ek1, epha5, epha7, epha8, ephb1, ephb2, etk2*
	Regulation of synaptic plasticity	22	*camk2b, cplx2, grik2, kcnn2, nf1, nptn, shank3, syngr1*
**Regulation of action potential**		21	*ank2, ank3, cacna1d, kcnip1, kcnc2, ryr2, scn1a, scn2b*
**Reg. of the neurotransmitter levels**		34	*cacna1a, gabra2, napb, nf1, rims1, slc6a13, snap25, stx1a, trpm7*

Several transcripts may originate from the same gene. This list is given an example, with no attempt to be exhaustive. See Additional files 5,6,7,8,9, and 10 for an enlarged version that include gene description and associated GO terms. *GO* Gene Ontology

### Metabolism

GO processes enriched within down-regulated genes were primarily related to metabolism (Fig. 3B, Additional file 11). A large number of these are implicated in biosynthetic processes (465 transcripts); peptide biosynthetic process (105 transcripts), regulation of translation (43 transcripts), carbohydrate derivative biosynthetic process (89 transcripts) and ATP biosynthetic process (20 transcripts) (Tables 3, Additional files 12 and 13). Other down-regulated biological processes included oxidation–reduction process (266 transcripts), catabolic process (184 transcripts), cell division (62 transcripts) and viral process (16 transcripts) (Fig. 3B, Additional file 14). Consistently, down-regulated gene programs included the ribonucleoprotein complex (166 transcripts), the mitochondria (217 transcripts)—including the mitochondrial respiratory chain complex I (12 transcripts), the mitochondrial matrix (29 transcripts), the catalytic complex (228 transcripts) and the oxidoreductase complex (29 transcripts) (Additional file 15).

**Table 3:** Examples of enriched gene ontologies and associated genes related to metabolism, viral process and cell division that were down-regulated (*p*-value ≤ 0.01) by ocean acidification in the olfactory rosette of F2 European Sea Bass (*Dicentrarchus labrax*) juveniles

GO term	Subcategory	Number of transcripts	Examples of regulated genes
**Peptide biosynthetic process**		105	*gtf2h2, mrpl35_37, mrps10-12, mrps21, noa1, ssr1, rs17, rpl18*
**Carbohydrate derivative biosynthetic process**	Tricarboxylic acid cycle	22	*aco1, aco2, cs, fh, idh1-3, mdh1, ogdh, pdhb, sdha-c, sucla2*
	Gluconeogenesis	9	*aldoc, eno2, pck2, fpb1, pgk1, slc25a11, slc25a12, smek2, tpi1a,*
	ATP biosynthetic process	20	*atp5l, atp5b, atp5g3, atp5a1, atp5i, atp5f1, atp5g1, atp5o*
**Oxidation–reduction process**		266	*aldh6a1, cyb5r3, cyb5r4, g6pd, gapdh, ndufs1-7, prdx1-6*
**Catabolic process**	Organic substance catabolic process	169	*afg3l1p, capn3, paox, pcsk9, psma6, tpp1, usp14, usp2a*
	mRNA catabolic process	37	*cnot11, ncbp1, ncbp2, pan2, pan3, patl1, smg5, smg8, uba52*
**Viral process**	Viral transcription	15	*rl23a, rl37a, rpl19, rpl23, rpl38-39, rps25, rps25, rps27, rps29*
**Cell division**		62	*ccna2, ccnb1-2, ccne1-2, cdc2, cdc20, cdc34, cdc40, cdk2, fzr1*

Several transcripts may originate from the same gene. This list is given as an example, with no attempt to be exhaustive. See Additional files 12,13, and 14 for an enlarged version that include gene description and associated GO terms. *GO* Gene Ontology

Finally, genes associated with AMP-activated protein kinase (AMPK) and mTOR signalling pathways, key energy-regulating pathways, were also differentially expressed between the two treatments. Specifically, mTOR signalling pathway genes (*mtor, lamtor1, lamtor3, mlst8*) were down-regulated while two AMPK subunit genes (*prkag1-2*) were found up-regulated by OA (Additional file 2).

### Innate immunity and anti-viral response

Transgenerational exposure to OA induced up-regulation of a substantial number of genes involved in innate antiviral immunity, partially listed in Table 4 and more exhaustively in Additional file 16. In particular, these belong to four classes of the germline-encoded Pattern Recognition Receptors (PRRs), which include nucleotide oligomerization and binding domain (NOD)-like receptors (NLRs), C-type lectin receptors (CLRs), toll-like receptors (TLR) and a retinoic acid inducible gene-I-like receptor (RLRs). Indeed, NLRs were the most represented PRRs with 109 transcripts, including three encoding protein nlrc3-like variants for which expression levels were elevated more than 30-fold (in Additional files 16 and 17, see transcript IDs: DLAgn_00244700, DLAgn_00265820 and DLAgn_00234590). Other up-regulated genes included the melanoma differentiation-associated gene 5 (*mda5* or *ifih1*) and Interferon-Stimulated Genes (ISG), which included GTPase-Immune-Associated Nucleotide-Binding Proteins (36 genes including *gimap4*, *gimap7* and *gimap8*), interferon-induced very large GTPase 1 (*gvinp1*) and GTP binding protein Mx (*mx*). Other up-regulated ISG transcripts included interferon-induced proteins with tetratricopeptide repeats (*ifit2*), MHC class I proteins, members of the TRIM protein family (trim25), an IFN-induced double-stranded RNA-activated protein kinase (*pkr1, prkrir*), the grass carp haemorrhagic virus (GCHV)-induced gene -1 (*gig1*), the nuclear autoantigen sp-100 (*sp100*), the sterile alpha motif and histidine/aspartic acid domain-containing protein 1 (*samhd1*), two apolipoproteins (*apo1,3*), a damage regulated autophagy modulator protein (*dram1*) and three caspases (*casp 1*, *casp 6* and *casp 7*). In addition, a small number of genes within the ISG category were also significantly down-regulated in the OA condition such as *gig2* or *irig, trim39* and *trim8*. While some transcripts associated with the interferon JAK-STAT signalling pathway were also up-regulated (*jak2, stat5.1, stat6*), several key genes in this pathway were down-regulated, such as mitochondrial antiviral signalling (*mavs*), myeloid differentiation factor 88 (*myd88*), tnf receptor-associated factor 3 (*traf3*) and several interferon regulatory factors (*irf4, irf5, irf6, irf8*).

**Table 4:** Categories and number of transcripts related to genes involved in innate immunity and/or antiviral defence up and down-regulated (*p*-value ≤ 0.01) by ocean acidification in the olfactory rosette of F2 European Sea Bass (*Dicentrarchus labrax*) juveniles

Category and Subcategory	Number of transcripts	Examples of regulated genes	Function
**PRRs**	109	*nlrc3; nlrc5; nlrp1b; nlrp4e; trpm2; nod2; nwd1; spry2; spry3*	pathogen recognition receptor; intracellular signal transduction [67, 68]
	8	*illr3; cd209; c209d*	pathogen recognition receptor/sensing RNA viruses [69]
	6	*tlr1l; tlr2-1; tlr21; tlr22; tlr3; tlr9*	pathogen recognition receptor/sensing RNA viruses [70–72]
	1	*ifih1*	pathogen recognition receptor/sensing RNA viruses [73]
**ISG**	36	*gimap7; gimap4; gimap8*	secretion of cytokines; anti-apoptosis of lymphocytes [74]
	9	*gvinp1*	Host resistance [75, 76]
	1	*mx*	antiviral effector; inhibition of viral replication [77–80]
	1	*ifit2*	inhibition of viral translation and replication [81, 82]
	13	Nd	immunomodulator; antigen presentation [83–85]
	30	*trim2,9,25,32,33; bty; btr01,06; mid2*	Sensors, restriction factors of viruses, regulators of IFN response [86, 87]
	12	*pkr1; prkrir; eif2ak2; thap4, 7; pak3,4*	inhibition of viral translation and replication [88]
	4	*samhd1*	Inhibition of viral replication [89]
	1	*sp100*	inhibition of viral replication [88]
	4	*apo1; apol3*	Degradation of the virus [88, 90, 91]
	1	*dram1*	Degradation of the virus [86, 88, 92]
	2	*casp1; casp7*	apoptosis effector [88]
**3**	*stat5.1; stat6; Jak2*		regulation of ISG expression [90, 93]
**22**	*il17a/f-1; il20; il34; il1r1; il20ra; il6st*		inflammation, protection against infection [94–96]
**15**	*c1ql2; c1ql4l; c1qtnf1; tnfsf15; cd40*		inflammation [97]
**7**	*mavs; myd88; traf3; irf4; irf5; irf6; irf8*		regulation of ISG expression [98–100]
**101**	*rps2, 12, 14; wdr12; rpf2; nip7; eif4; eif4ebp3l; eif4a3; eif4ea; eif4b*		inflammation, viral translation [98–100]

*PRR* germline-encoded Pattern Recognition Receptors, *ISG* Interferon (INF)-stimulated Genes, Several transcripts may originate from the same gene. This list is given as an example, with no attempt to be exhaustive. See Additional files 15,16, and 17 for an enlarged version that include gene description and associated GO terms. *Nd* Not defined

Finally, a large swath of cytokine-encoding transcripts were up-regulated, including 22 interleukin-associated genes (e.g. *il34; il20; il17a/f-1; il1rl2; il6st; il20ra; il18r1; il7r; il4r*) and 15 tumour necrosis factor-associated genes (*tnip2; c1ql2; c1ql4l; c1qtnf1; tnfsf15; cd40*) (Table 4, Additional file 5). In contrast, almost 100 transcripts related to genes typically involved in viral protein translation during infection (e.g. *rps2, 12, 14; wdr12; rpf2; nip7; eif4; eif4ebp3l; eif4a3; eif4ea; eif4b*) were down-regulated (Table 4, Additional file 5).

## Discussion

Because the olfactory epithelium in fish is in direct contact with water, its cells are particularly exposed to environmental changes. However, in the context of global climate change and its longer-term impact of marine life, it is difficult to predict how fish and other species will acclimate and adapt to an increasingly acidic marine environment. In this study, we experimentally investigated transgenerational long-term consequences of OA on the olfactory epithelium transcriptome in F2 *D. labrax.* Specifically, we sought to understand how predicted end-of-century OA alters the olfactory system in a way that reflects long-term acclimation in F2 offspring, and how such changes might impact on overall robustness, such as survival after infection, a common problem in aquaculture. It is noteworthy that although parents were also exposed lifelong to OA, our experiment does not allow to disentangle whether the impacts observed in the offspring could have been carried-over from their parents, and if so, if they were restored, improved or worsen in the F2. Cumulatively, we identified ~ 9,000 OA-induced differentially expressed transcripts, representing roughly a third of the total number of transcripts (34%), that revealed a profound modification of the *D. labrax’* olfactory epithelium transcriptomic profile.

**Fig. 4:**
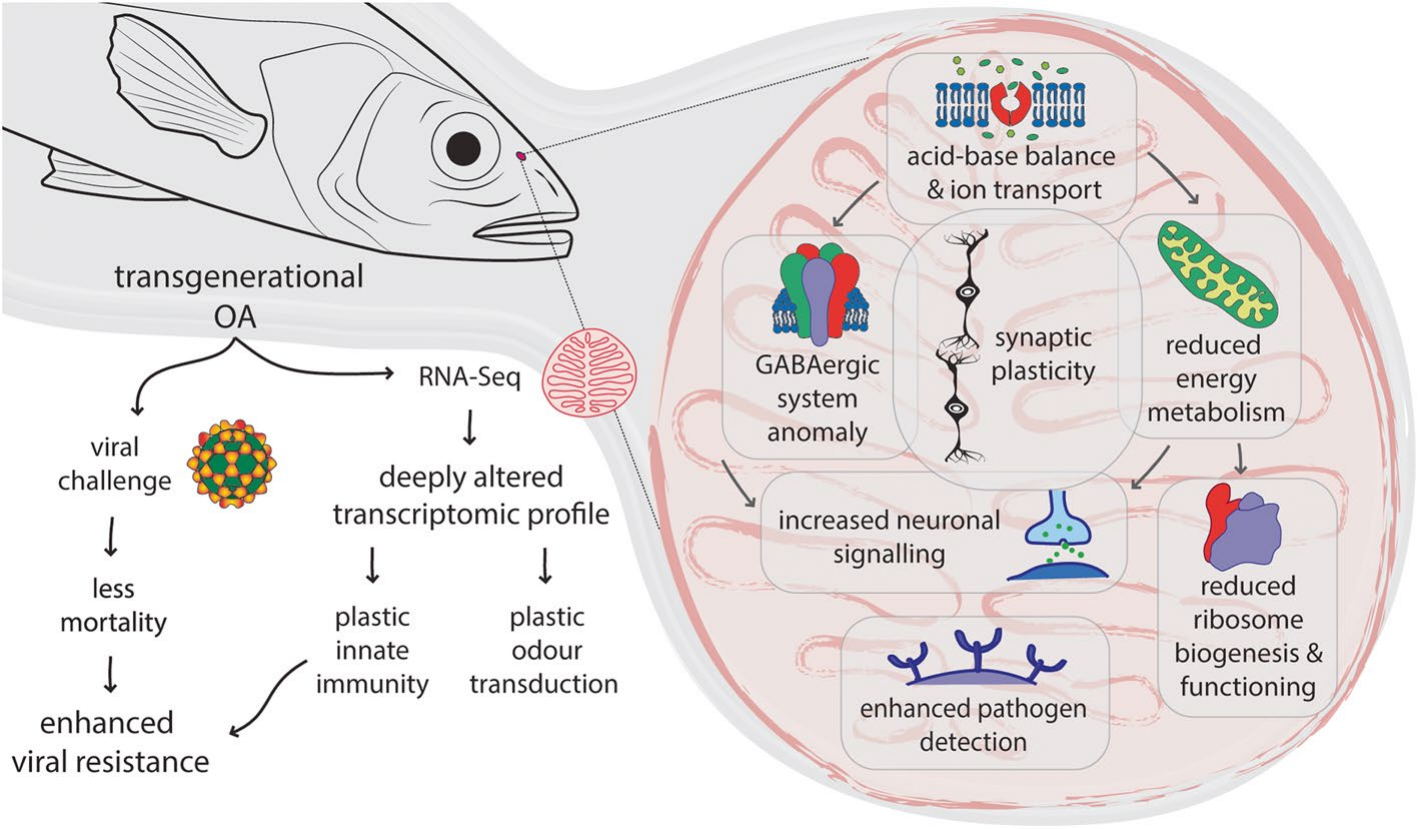
Conceptual framework. The impacts of transgenerational ocean acidification (OA) on F2 *Dicentrarchus labrax* juveniles are shown for both the viral challenge and the RNA-Seq analysis

Impacts of olfactory systems in the context of OA were reported to reverberate on olfactory behaviour in broadly related work [30, 32, 38, 101, 102]. For instance, *Dicentrarchus labrax*, when exposed to predicted end-of-century OA for up to two weeks, experienced declined capacity for detecting food sources and avoiding predator-associated scents [30]. This was related to a down-regulation of the AMPA glutamate receptors *gria1b* and of *camk2* involved in synaptic plasticity, and to an up-regulation of *tmub1* involved in AMPA receptor cycling in the olfactory bulb. Conversely, our data revealed an up-regulation of several genes implicated in synaptic plasticity (*camk2*, *nptn*, *syngr1*, AMPA and NMDA receptors). In the same way, while genes responsible for maintaining cell excitability (*scn4* and *cacna2*) and neuronal growth (*efnb2a*) were down-regulated after short-exposure to OA [30], we found them up-regulated together with other ion channels (*scn1a*, *scn8a*, *cacna1*, *cacng3*, *tmem37*) and neuronal growth factors (*negr1*, *fgfr2*) after long-term transgenerational OA-exposure in the F2. The present increase in the transcription of genes involved in synaptic plasticity, signal transduction (including the GABAergic pathway), neuron activity, excitability and wiring in the olfactory rosette of F2 *D. labrax* (Fig. 4) which differs from previous data observed in the same species might be explained by the duration of exposure.

The up-regulation of genes from the solute carrier (SLC) 4 gene family involved in ${Na}^{+}/{HCO}_{3}^{-}$ cotransport (*slc4a7*, *slc4a8*, *slc4a10*) that we found in the olfactory epithelium of F2 juveniles may result from compensation of the intracellular influx of ${HCO}_{3}^{-}$ to cope acidosis [24, 25, 30]. Our data indicate a transgenerational maintenance of this mechanistic compensation which has been previously associated with a dysfunction of the GABAergic neuronal system in F1 generation [e.g. 24]. Furthermore, OA-induced up-regulation of numerous actors of the GABA signalling pathway (e.g. *cacna1a*) is similar to the within generation response observed in the brain of spiny damselfish suggesting that GABAergic dysfunction also occurs after two generations of OA-exposure in *D. labrax* [103].

Whether the long-term plasticity that we observed in the present study concerning genes involved sensory system lead to either maintaining olfactory sensitivity or not, is an open question that needs electrophysiological measurement and/or whole-animal olfactory behavioural experimentation. Electrophysiological measurement may help to understand if regulation of neuronal activity associated to transgenerational exposure to OA is related to an amplification of the olfactory response facing odorants that have, at their turn, underwent structural and charge distribution changes due to OA [31, 32].

We also observed transcriptome changes indicative of a metabolic depression in the olfactory epithelium, as exemplified by down-regulation of genes involved in ATP synthesis*,* the mitochondrial electron transport system (*cox* genes) and genes involved in energetically costly processes (e.g. macromolecules biosynthesis). At a general level, we also detected higher expression of genes related to the AMPK signalling pathway, consistent with related work in the brain of fish exposed to OA [103]. Since AMPK is suited to participate in metabolic depression in response to environmental energy stress, the up-expression of AMPK related genes observed in the present study is consistent with a metabolic depression in the cells of olfactory epithelium [104]. In fish, as in other vertebrates, oxidative metabolism mediates neuronal activity and survival [105]. In the light of this, we presume that the OA-induced stimulation of AMPK signalling pathway observed in the present study may result from the necessity for olfactory neurons to manage the highly energy demanding processes of acid–base regulation to cope intracellular acidosis and guarantee the correct functioning of neuronal activity and olfactory function. These additional energy requirement for acid–base regulation is unlikely to induce a metabolic depression of the whole animal metabolism in F2 juveniles. This is supported by studies of heart mitochondrial metabolism [65] and measurement of whole animal metabolic rate (Howald et al., in prep. / FC Mark, pers. obs.) that did not observe differences between control and OA exposed individuals in the same cohort of fish. Again, additional experimentation is needed to identify whether the present regulation of genes involved in metabolic homeostasis is associated to disruption of neuronal activity and can ultimately impact whole-animal olfactory function.

Extending from these findings, and unexpectedly, we also discovered that *D. labrax*, upon transgenerational exposure to OA, up-regulated gene programs conferring elevated resistance to virus infection. Consistent with these molecular changes, we found in our betanodavirus infection study that the OA-exposed F2 cohort succumbed to infection later and that fewer fish died than control F2 fish (Fig. 2). Across different organs in OA-exposed and control fish, virus presence and concentrations were similar, indicating that the viral infection in and of itself was similar, and that the difference in disease resistance was due to a superior innate defence relative to fish exposed to OA. It is noteworthy that high seropositive rates in both treatments suggest that specific immune capacity was not affected and that the difference observed in the seropositive rates between treatments was probably due to the difference of mortality rate and the secondary reinfections associated by dead fish.

In related work, Bresolin de Souza and collaborators found that a three-month exposure to OA in Atlantic juvenile halibuts was associated with increased activity of complement component C3, lysozyme and fibrinogen, known factors in innate and complement systems though antiviral and inflammatory activities [52, 53]. Apart from this work, OA effects linked to immune function has been reported for shellfish [47–49], but our insight into long-term acclimation for marine fish, and their potential ramifications for aquaculture remain largely unknown [106]. Such knowledge is urgently needed in a progressively acidic marine environment. Here, we reveal for the first time that fish may acquire an improved capacity to resist a viral infection upon years of transgenerational exposure to OA. In our transcriptomic characterization of the olfactory epithelium we detected up-regulation of gene programs linked to innate antiviral activity; pathogen receptors genes, Interferon-Stimulated Genes (ISG) and ribosome-related genes.

Detection of an invading pathogen is a critical first step in the initiation of a robust immune response (uncapped viral RNA/DNA and/or cell wall components) through Pattern Recognition Receptors (PRRs) [107]. In the OA-exposed F2 cohort, we detected a robust up-regulation of four out of the five types of PRRs relative to control F2 fish, suggesting an enriched extent of pathogen detection as a first contingency response upon viral invasion. Of note, these are germline-encoded genes, suggesting that their up-regulation can partly result from trade-offs in parents carried over to offspring, although our analysis does not allow us to distinguish this possibility from solely F2 generation plasticity. Of the up-regulated PRR classes, nucleotide oligomerization and binding domain (NOD)-like receptors (NLRs) NLRs were the most abundant. NLR can act as sterile and intracellular pathogen stress signal receptors and are therefore poised to play a central role in the inflammatory response [90, 108] and homeostasis of microglia, the only central nervous system immune cells present in fish [109, 110].

The second PRR class, C-type lectin receptors (CLRs), mediate bacteria- and fungi-associated responses, but following viral recognition, can induce both protective and detrimental effects depending on the pathogen [111]. Members of the mannose receptor, the asialoglycoprotein receptor and c-type lectin domain families, were represented among the up-regulated CLR genes (Additional file 2). Interestingly, the melanoma differentiation-associated protein 5 (*mda5* or *ifih1*) gene, which encodes for a member of RLRs and several members of the TLRs gene family, both considered as key virus sensors were also up-regulated [111, 112]. Stimulation of MDA5 or TLRs by RNA viruses is known to result in type I interferon response activation [70].

Interferons are signalling cytokines secreted by every type of cell in order to interfere with viral progression by regulating the expression of more than 1000 genes at the transcriptional level [113, 114]. These genes are called Interferon-Induced Genes (ISG) and exhibit a wide array of antiviral properties [93, 115]. A large proportion of orthologous ISGs have been identified in fish and humans and very few fish ISG have no human ortholog (e.g. gig1, gig2, vig-B319 [116]). The mechanisms of antiviral activity of fish-specific ISGs remain poorly understood [90, 117]. Here, we found that OA-exposed fish expressed several up-regulated ISGs whose functions may partially explain their enhanced resistance to viral infection. These include the IFN-induced double-stranded RNA-activated protein kinases (*pkr1, prkrir*), a particular ISG subset that functions as PRR, antiviral effector, and inhibitors of virus translation and replication combined [90, 117, 118]; genes from the GTPase imap family (*gimap8, 4, 7*), known to mediate cell-autonomous resistance against pathogens [77, 113, 119]; and genes from the TRIM family (*trim16, 39*) that play pivotal role in viral restriction, modulation of immune signalling, autophagy and formation of cellular structures [120]. The regulation of ISG transcription can take place via the classical interferon mediated JAK-STAT signalling pathway and/or through a variety of non-canonical pathways independent of interferon induction [93, 121, 122]. Here, we found that genes involved in the JAK-STAT pathway were both up- and down- regulated in the OA-exposed fish cohort. To better understand ISG signalling pathway regulation in the context of viral infection in fish, analyses at the post transcriptional levels (e.g. phosphorylation status) for JAK-STAT and non-canonical pathways would be necessary.

Finally, we found that transgenerational exposure to OA triggered a down-regulation of genes coding for ribosome biogenesis, ribosomal proteins and eukaryotic translation initiation factors, indicative of reduced ribosome abundance and malfunction. This is confirmed by the GO analyses that show strong down-regulation of both translation and macromolecule biosynthetic process (Fig. 3B). During an infection, the virus hijacks the host ribosomes to produce new viral particles along with host cellular factors to initiate viral translation [123, 124]. We hypothesised that as OA induced a malfunction of the host’s ribosomes, viruses may lack the biosynthetic machinery to translate and transcribe their nucleic acids, which is supported by the downregulation of viral transcription and processes (Fig. 3B). This, together with the up-regulation of PRRs and ISGs might explain the observed superior resistance during the viral challenge.

VNN outbreaks are considered one of the most relevant infectious constraint for the culture of a variety of fish species. Hence, the new insights we provide into how a marine teleost of economic interest undergoes transgenerational acclimation could be of precious concern to aquaculture. Although juvenile acclimated to transgenerational OA were more resistant to VNN, based on the changed transcriptome, their metabolic and odour transduction programs were altered, which may in turn modify the olfactory perception of a wide array of chemical cues that may consequently impact odour-mediated behaviours including feeding, homing and other inter- or intra-specific interactions such as sociability, mating, competition and predator detection (Fig. 4). Understanding how the interplay between acidification and warming over generations modulate olfactory behaviour and viral resistance could be useful to develop new strategies for maintaining the health and production in aquaculture facilities for either commercial or scientific purposes.

## Conclusions

We report here that transgenerational exposure to OA induces a deep modification of the transcriptomic profile in the olfactory epithelium of the *D. labrax* that include plastic responses related to ion balance and transport, neuronal activity and plasticity, energy metabolism and innate immunity (Fig. 4). This transgenerational plasticity may be considered as an acclimation (adaptive plasticity) to prevent more severe OA-induced physiological disruption at the whole organism level [103, 125]. It is noteworthy that immune system factors such as cytokines, interferons and interleukins also play a role in central nervous system and brain development, and can induce changes in neural network activity, supporting the intricate interplay between the immune and the nervous system [126, 127]. Additional experimentations based on electrophysiology and behavioural tests would help determine whether the regulation that we observed in the neuronal plasticity and activity gene programs are associated with a perturbation of the olfactory function, as suggested by the regulation of key processes associated with energy metabolism. Likewise, further studies will be necessary to more globally characterize OA-induced effects on immune status in both cultured and wild fish and its capacity to resist the most fish pathogens in a changing ocean.

## Methods

Animal experiments in this study were conducted following the European Commission recommendation 2007/526/EC and Directive 2010/63/EU for the accommodation and care of animals used for experimental and other scientific purposes. The OA conditioning was performed at the French Research Institute for Exploitation of the Sea (IFREMER) in Plouzané, within the facilities of the Laboratory of Adaptation, Reproduction and Nutrition of fishes (ARN) (Agreement number: B29-212–05). The viral challenge was performed at the French Agency for Food, Environmental and Occupational Health & Safety (ANSES) in Plouzané (Agreement number: D29-212–3). Each procedure was the subject of a specific authorization issued by a French Ethics Committee for animal testing (APAFIS #2,018,032,209,421,223 for OA conditioning, and APAFIS #202,001,161,613,768 for viral challenge).

### Animal husbandry and experimental setup

F2 juveniles (18 months old, non-sexually differentiated) of the European sea bass *Dicentrarchus labrax* used in the present study originated from an in vitro fertilisation of 4 years old F1 parents exposed from larval to adult stages to control conditions (~ pH8.0) or to OA conditions (~ pH7.6) [54, 63–65]. Sperm and eggs from the F1 were collected and pooled from 20 males and 6 females of each pH-treatment (see methodological summary in Fig. 1). Luteinizing hormone releasing hormone (LHRH) was injected to stimulate synchrony in oocytes full maturation. Taking great care of maintaining parental pH conditions, eggs were hatched and the resultant F2 offspring were reared in water at the same pH as their parents. Rearing conditions during larval and juvenile stages were similar to those described in previous studies [64–66]. For both treatments, seawater temperature and salinity followed seasonality of the Bay of Brest. 402 juveniles (201 per treatment) were distributed evenly in 6 culture tanks (400 L, three tanks = three replicas per treatment) that were part of an open-circuit system. To guarantee high quality, seawater pumped 500 m off the coastline at a depth of 20 m passed through a sand filter, a tungsten heater, a degassing column packed with plastic rings, a 2-μm filter membrane, and a UV lamp. Seawater for the control treatment was then poured into each of the three replicas tanks. Seawater for the OA treatment was injected with CO_2_ at constant flow (through manipulation of a flowmeter (Aalborg, USA) connected to a CO_2_ bottle (Air Liquide, France)) in a header tank equipped with a degassing CO_2_ column to favour mixing. Then, low pH seawater was poured into each of the 3 replica tanks. pH in NIST scale and temperature in the six tanks were daily measured with a WTW 3110 pH meter (Xylem Analytics Germany, Weilheim, Germany; with electrode: WTW Sentix 41) calibrated daily with pH4.0 and pH7.0 buffers (WTW, Germany). Total alkalinity was measured once a week following the adapted protocol of Strickland and Parsons [128]: a 50 ml sample of filtered tank seawater was mixed with 15 ml HCl (0.01 M) and pH was measured immediately. Total alkalinity was then calculated with the following formula:

$$TA=\frac{{V}_{HCl}*{C}_{HCL}}{{V}_{sample}}-\frac{\left({V}_{HCl}+{V}_{sample}\right)}{{V}_{sample}}*\frac{\left\{{H}^{+}\right\}}{y{H}^{+}},\left[\frac{mol}{l}\right]$$

with, total alkalinity (TA, mol l^−1^), volume (V, l) of HCl or of the sample, concentration (C, mol l^−1^) of HCl, hydrogen activity (H^+^, 10^−pH^) and hydrogen activity coefficient (УH^+^, here = 0.758). The software CO2SYS using the constants from Mehrbach et al. refitted by Dickson and Millero [129–131] were used to calculate the carbonate chemistry components. Summary data is available in Table 5 for both the F1 and the F2 and the associated partial raw data in available in the SEANOE repository [132]. Fish were fed ad libitum with diets that meet their nutritional requirements (Vitalis Cal, Skretting, Stavanger, Norway). No significant difference was observed in the mean body weights (t test, t = 0.02, df = 64.52, *p*-value = 0.98) of the 18-months-old juveniles between the two treatments (*n* = 35). F2 juveniles from the two treatments behaved and fed in a relatively similar way. Mortality events were not detected.

**Table 5:** Summary of the sea water physico-chemical parameters averaged on the data period (mean ± se) for both the F1 and F2

	F1: 53-months-old parents^*^		F2: larvae^**^		**F2: 18-months-old juveniles**	
**Rearing period**	24 October 2013 – 26 March 2018		1 April 2018 – 1 June 2018		2 June 2018 – 17 October 2019	
**Available Data period**	8 February 2016 – 6 March 2018		1 April 2018 – 1 June 2018		2 June 2018 – 12 February 2019	
**Sea Water Parameter**	**Control Treatment**	**Acidified Treatment**	**Control Treatment**	**Acidified Treatment**	**Control Treatment**	**Acidified Treatment**
Salinity	33.6 ± 0.3	33.6 ± 0.3	31.8 ± 0.1	31.8 ± 0.1	33.0 ± 0.2	33.0 ± 0.2
O2 (%)	91.4 ± 1.5	92.1 ± 1.3	94.2 ± 1.5	94.2 ± 1.5	92.4 ± 0.7	94.3 ± 0.6
Temperature (°C)	14.1 ± 0.6	14.1 ± 0.6	15.2 ± 0.05*	15.5 ± 0.05*	16.1 ± 0.3	16.3 ± 0.3
pH_NIST_	7.99 ± 0.02	7.62 ± 0.01	8.12 ± 0.01*	7.67 ± 0.02*	7.98 ± 0.02	7.64 ± 0.01
pH_Total_	7.88 ± 0.02	7.50 ± 0.04	7.98 ± 0.02	7.46 ± 0.03	7.86 ± 0.02	7.53 ± 0.01
TA **(**μmol kg_SW_^−1^**)**	2401 ± 49	2409 ± 46	2360 ± 39	2330 ± 39	2376 ± 20	2780 ± 18
pCO2 (μatm)	672 ± 41	1677 ± 54	503 ± 32	1867 ± 129	694 ± 26	1598 ± 4

*TA* total alkalinity, *pCO*_*2*_ CO_2_ partial pressure, Mean ± se calculated on daily data (61 days). Partial raw data available in [132]

^*^ Average sea water parameters based on monthly measurements

^**^ Average sea water parameters based on weekly measurements

### RNA extraction

Prior to sampling, fish were fasted for 24 h. Then, 18-months-old juveniles were first anesthetized (20 mg L^−1^), and then euthanized with a lethal dose (200 mg L^−1^) of tricaine methane sulfonate 222 (MS222, Pharmaq, Fordingbridge, Hampshire, UK). Olfactory rosettes were collected from 7 individuals per treatment and quickly stored in RNA Stabilization Reagent (RNAlater, Qiagen, Hilden, Germany) following recommendations from the supplier. Total RNA was extracted using Extract-All reagent (Eurobio, Courtaboeuf, Essonne, France) combined with Nucleospin RNA column that includes one step of DNase treatment (Macherey–Nagel, Düren, Germany) according to the manufacturer’s instructions. The concentration and purity of extracted RNA were verified (260/280 ratio > 2) using an ND-1000 NanoDrop® spectrophotometer (Thermo Scientific Inc., Waltham, MA, USA). The integrity of RNA was checked by electrophoresis using an Agilent Bioanalyzer 2100 (Agilent Technologies Inc., Santa Clara, CA, USA). All samples showed an RNA integrity (RIN) score > 9. RNA samples were stored at − 80 °C for further RNA sequencing.

### RNA-sequencing analysis

RNA samples extracted from the olfactory rosettes were sent to the sequencing platform GenomiX (MGX, Montpellier, France) for transcriptome analysis through RNA sequencing (RNA-Seq).

#### cDNA libraries

Libraries of cDNA were constructed using TruSeq Stranded mRNA kit (Illumina, San Diego, CA, USA) according to the manufacturer’s instructions. Briefly, poly-A RNAs were purified using oligo-d(T) magnetic beads from 863 ng of total RNA. Poly-A RNAs were fragmented and underwent a reverse transcription using random hexamers. During the second strand generation step, dUTP substituted dTTP in order to prevent the second strand to be used as a matrix during the final PCR amplification. Double stranded cDNAs were adenylated at their 3' ends and ligated to Illumina's indexes. Ligated cDNAs were amplified following 15 PCR cycles. PCR products were purified using AMPure XP Beads (Beckman Coulter Genomics, Brea, CA, USA). Libraries were validated using Standard Sensitivity NGS kit on Fragment Analyzer (Agilent Technologies, Santa Clara, CA, USA).

#### Libraries sequencing

Fourteen libraries were pooled in equimolar amounts. The balance between all samples of the pool was assessed by using KAPA Library quantification kit (Roche, Bâle, CHE). The pool was then sequenced on a Novaseq 6000 (Illumina, San Diego, CA, USA) on a SP flow cell in single-read 100 nt mode according to the manufacturer’s instructions. This sequencing produced between 40 and 50 million passed filter clusters per library.

#### Sequencing quality control

Image analyses and base calling were performed using the NovaSeq Control Software and Real-Time Analysis component (Illumina, San Diego, CA, USA). Demultiplexing and trimming were performed using Illumina's conversion software (bcl2fastq 2.20). The quality of the raw data was assessed using FastQC from the Babraham Institute and the Illumina software SAV (Sequencing Analysis Viewer). FastqScreen was used to estimate the potential level of contamination.

### Transcriptomic and gene ontology (GO) analysis

Raw reads mapping and gene quantification were done using STAR aligner (v2.7.2c) [133] to the *D. labrax* reference genome guided by the reference gene annotation [134]. Differential expression analysis was performed with the DESeq2 package (Bioconductor) [135] using an adjusted *p*-value cut-off of 0.01. The SRR accession numbers for the raw sequence data are SRR15222852-65. *Cbln11* structural annotation was carried out manually in GenomeView [136] based on BlastHit of *Cbln11* sequences available at NCBI and from mapped RNA-Seq reads. Gene structure was exported in *gtf* format and added to the current annotation. The datasets supporting the conclusions of this article are available in the SRA repository under accession numbers: SRR15222859-65 (https://www.ncbi.nlm.nih.gov/sra/?term=SRR15222852) and SRR15222852-58 (https://www.ncbi.nlm.nih.gov/sra/?term=SRR15222859).

To extract biological meaningfulness and to visualize potentially affected pathways, a Gene ontology (GO) enrichment analyses was performed separately on significantly up- or down-regulated genes under OA. Raw reads from RNA-Seq were imported into Galaxy instance of Ifremer [137]. An obo GO file and the product annotation file for *D. labrax* [134] were used to analyse significantly (*p* < 0.01) up or down-regulated genes. FDR was corrected with the Benjamini–Hochberg test. The corrected *p*-value to apply to the graph output was set to 0.01.

### Viral challenge experiment

Betanodavirus strain W80 isolated from diseased *D. labrax* displaying typical signs of VNN was used in this study. According to Castri et al. [138], a stock of virus was produced at 24 °C on the SSN-1 (Striped Snakehead fish; *Ophicephalus striatus*) cell line (L15 medium, 10% FBS, pH7.6) and frozen. Cell debris was removed by centrifugation for 15 min at 2000 g; the virus was then aliquoted and stored at − 80 °C. Before the challenge, viral titration was carried out on one of the aliquots after a single freeze–thaw cycle based on the tissue culture infectious dose technique (TCID_50_) described by [139]. The infectious titer of the viral production was calculated according to the method of Kärber [140], and was found to be 1 × 10^8^ TCID_50_/mL.

After 18-months rearing of the F2, 148 juveniles per treatment were divided into four flow through tanks of 400L (Fig. 1) and were challenged by immersion in a 3-h bath with W80 at 25 °C and natural pH to mimic the environmental conditions where the clinical signs of disease were reported. For the viral challenge, the water flow was interrupted, the oxygenation was increased and the water volume was reduced to 100 L. Three out of four tanks per treatment were exposed for 3 h with an infectious dose of 5.10^4^ TCID_50_/mL^−1^ of W80. The fourth tank per treatment was exposed in the same conditions to SSN-1 cell supernatant free from the virus and used as a negative control without virus. After a 3-h bath exposure, the water flow was restored to slowly dilute the virus titration. The system was maintained open and the water temperature (25 °C ± 2 °C) was continually measured and recorded with a wireless probe (Cobalt, Oceasoft®) coupled to an acquisition system (ThermoClient 4.1.0.24). Juveniles were fed once a day with commercial pellets (Neo Start Coul 2 from Le Gouessant Aquaculture) except on the day of the viral infection.

Mortality was recorded twice a day during 45 days post-infection (dpi). Dead fish were stored at − 20 °C until viral examination. Virus concentrations were determined on a pool of organs (brain and eyes) from six fish per treatment that died at the peak of mortality (9 dpi in the control treatment; 11 dpi in OA treatment) and in five survivor fish at the end of the challenge by immunofluorescence assay on the SSN-1 and E11 cell lines according to the adapted protocol by Dussauze et al. [139]. The detection of anti-VNNV antibodies in *D. labrax* plasma was performed in survivor fish (23 control and 26 OA-exposed fish) using an ELISA test [139].

Survival curves were estimated using the Kaplan–Meier method and were compared by a log rank analysis using the online platform BiostaTGV. Firstly, survival curves for each replica within each treatment were compared, and if no significant difference was observed, results from the 3 replicas per treatment were pooled. In case of significant difference, each replica was analysed separately and compared to the other treatment. 

## Supplementary Information

**Additional file 1.** Matrix of normalized read counts for the 14 libraries from either the acidified treatment (A) or the control treatment (C). Eight samples of each treatment were taken but only seven were used for further analysis.

**Additional file 2.** Full list of differentially expressed genes (p-adj ≤ 0.01) of the first generation (F1) of European Sea Bass (*Dicentrarchus labrax*) exposed for 2 years to intergenerational ocean acidification (OA; F0: 4-year OA-exposure). In the Gene symbol column, the * represent excel errors due to conversion into dates (Abeysooriya et al., 2021) while - indicates the absence of an annotated Gene symbol. FC: fold change expressed as Log2 FC. ↑: upregulated genes; ↓: downregulated genes. P-adjusted are p-values corrected for FDR with the Benjamini-Hochberg method. ns: not significant. In the colum GO (Gene Ontology) term, F: molecular function, P: biological process; C: cellular component.

**Additional file 3.** Genes involved in chloride homeostasis, bicarbonate transport and regulation of pH that are regulated (*p* value ≤ 0.01) by OA exposure.

**Additional file 4.** Cell component Gene Ontologies enrichment (*p*-value ≤ 0.01) within the genes that were up-expressed (*p*-value ≤ 0.01) by OA.

**Additional file 5.** Genes involved in neuron projection (including axon guidance and extension) up-expressed (*p*-value ≤ 0.01) in OA group.

**Additional file 6.** Biological processes Gene Ontologies enrichment (*p*-value ≤ 0.01) within the genes that were up-expressed (*p*-value ≤ 0.01) by OA.

**Additional file 7.** Genes involved in the neurotrophin TRK receptor signalling pathway, glutamate signalling pathway and ephrin signalling pathway that were up-expressed (*p*-value ≤ 0.01) by OA.

**Additional file 8.** Genes involved in the regulation of synaptic plasticity that were up-expressed (*p*-value ≤ 0.01) by OA.

**Additional file 9.** Genes involved in the regulation of action potential that were up-expressed (*p*-value ≤ 0.01) by OA.

**Additional file 10.** Genes involved in the regulation of the neurotransmitter levels that were up-expressed (*p*-value ≤ 0.01) by OA.

**Additional file 11.** Biological processes Gene Ontologies enrichment (*p*-value ≤ 0.01) within the genes that were down-expressed (*p*-value ≤ 0.01) by OA.

**Additional file 12.** Genes involved in peptide biosynthesis and regulation of translation that were down-expressed (*p*-value ≤ 0.01) by OA.

**Additional file 13.** Genes involved in tricarboxylic acid cycle, gluconeogenesis and ATP biosynthetic process that were down-expressed (*p*-value ≤ 0.01) by OA.

**Additional file 14.** Genes involved in oxidation reduction process, organic substance catabolic process, mRNA catabolic pathway, cell division and viral process that were down-expressed (*p*-value ≤ 0.01) by OA.

**Additional file 15.** Cell Component Gene Ontologies enriched (*p*-value ≤ 0.01) within the genes that were down-expressed (*p*-value ≤ 0.01) by OA.

**Additional file 16.** Expanded version of Table 2 showing some significantly (p ≤ 0.01) differentially expressed genes related with innate antiviral immunity (not rigorous list) of the first generation (F1) of European Sea Bass (*Dicentrarchus labrax*) exposed for 2 years to intergenerational ocean acidification (OA; F0: 4-year OA-exposure). PRR: germline-encoded Pattern Recognition Receptors; ISG: Interferon-Stimulated Genes. FC: fold change expressed as Log2 FC. ↑: upregulated genes; ↓: downregulated genes. P-adjusted are p-values corrected for FDR with the Benjamini-Hochberg method. In the colum GO (Gene Ontology) term, F: molecular function, P: biological process; C: cellular component. Note: not every gene sequence possess an annotated Gene symbol. 

**Additional file 17.** List of the first 20 genes showing the highest OA-induced up-expression levels (*p*-value ≤ 0.01).

## Data Availability

The datasets generated and analysed in this article are included within the article as additional files 1 to 17 or are available in the SEANOE repository (https://doi.org/10.17882/87395) and in the SRA repository (https://www.ncbi.nlm.nih.gov/sra/?term=SRR15222852 and https://www.ncbi.nlm.nih.gov/sra/?term=SRR15222859).
