# Correction to: The extensive transgenerational transcriptomic effects of ocean acidification on the olfactory epithelium of a marine fish are associated with a better viral resistance

**DOI:** 10.1186/s12864-023-09299-0

**Published:** 2023-05-26

**Authors:** Mishal Cohen‑Rengifo, Morgane Danion, Anne‑Alicia Gonzalez, Marie‑Laure Begout, Alexandre Cormier, Cyril Noel, Joelle Cabon, Thomas Vitre, Felix C. Mark, David Mazurais

**Affiliations:** 1 IFREMER, PFOM‑ARN, Plouzane, 29280 France; 2 Ploufragan‑Plouzane Laboratory, Fish Viral Pathology Unit, French Agency for Food, Environmental and Occupational Health and Safety (ANSES), Technopole Brest-Iroise, Plouzane, 29280 France; 3 MGX, CNRS, INSERM, University of Montpellier, Biocampus Montpellier, Montpellier, France; 4 MARBEC, University of Montpellier, CNRS, IFREMER, IRD, Palavas‑les‑Flots, 34250 France; 5 IFREMER, SEBIMER, Plouzane, 29280 France; 6 Alfred Wegener Institute, Department of Integrative Ecophysiology, Helmholtz Centre for Polar and Marine Research (AWI), 27570 Bremerhaven, Germany

**Correction:** ***BMC Genomics*** **23, 448 (2022)**

10.1186/s12864-022-08647-w

Following the publication of the original article [1], it was noted that due to a typesetting error the Additional Files 1–17 did not display correctly.

The original article [1] has been updated.
